# Recycling of Flexible Plastic Films: Emergent Technologies

**DOI:** 10.3390/polym18162031

**Published:** 2026-08-21

**Authors:** Jacob S. Licht, Marina Tsianou, Paschalis Alexandridis

**Affiliations:** Department of Chemical and Biological Engineering, University at Buffalo, The State University of New York (SUNY), Buffalo, NY 14260-4200, USA

**Keywords:** flexible film, multilayer film, delamination, polymer separation, plastics recycling, mechanical recycling, molecular recycling, chemical recycling, sustainability, circular economy

## Abstract

Plastic is a valuable material for packaging of food and pharmaceuticals, protective wrappings in construction and agriculture, and fluid storage. Flexible plastic or plastic film waste from packaging, agriculture, and construction applications grows at a rate of at least 92 million metric tons a year, is considered challenging to recycle, and is typically landfilled. In recent years, there have been great advancements in plastic recycling technology in order to deal with the global challenge of plastic waste buildup and support legislation from a local to national level to implement recycling. This work highlights the most recent advancements in plastic film recycling. Plastic films are mono- or multilayered based on what their applications will be, with multilayer multimaterial films being the more challenging feedstock for recycling. Mechanical recycling cannot easily process flexible films. Pyrolysis can use polyolefin-based film as feedstock but is not practiced at scale to match the rate of plastic film waste generation, and incineration can recover energy from film feedstock but is not recycling plastic. This has motivated the development of new recycling technologies designed around plastic films. Better characterization technologies to identify film compositions in municipal waste streams have been key to sorting out film feedstock for mechanical recycling and the baling of flexible plastic waste, but they struggle with multilayer films and black plastic. Compatibilization enables the recycling of mixed plastic waste but requires polymer compositions for selecting specific compatibilizers. Dissolution–precipitation recovers individual types of polymers from multilayer films and, at the same time, can purify polymers from additives or contaminants, but requires intense solvent processing and associated energy. Delamination of multilayer films can separate and recover solid films of polyolefins at relatively low amounts of solvent but requires quality feedstock to be efficient. Both dissolution–precipitation and delamination recycling of films recover the original polymer molecules and maintain their embodied energy, hence support circularity. In the case of PET-containing films, depolymerization to recover PET monomers offers opportunities to recycle challenging film feedstock.

## 1. Introduction

Consumer demand for plastic materials has driven plastic production with a high market value, especially in packaging applications. Flexible plastics are primarily used in packaging products [1], with food and medical packaging being key consumers of multilayer films, leading to a predicted market value of 456 billion USD by 2027 [2], and constituting 0.5% of the United States (US) Gross Domestic Product (GDP) in 2025 [3]. Should plastic production growth continue, up to 1.1 billion tons of plastic may be produced by 2050, with 98% being made from fossil fuels with virgin plastics [4]. By 2024, global plastic production had reached 430 million metric tons (MMT) [5,6]. Single-use plastics, 98% of which come from feedstocks made of fossil fuels, reached 139 MMT consumption by 2021, while recycling capacity was 114 MMT lower than (i.e., severely lagging) the production rate of single-use plastics at the time [4]. By 2024, approximately 40% of plastics are attributed to single-use plastics, primarily in packaging applications [7], equivalent to 172 MMT [5,7] in 2024. This furthers the gap between single-use plastic production and recycling by an additional 17 MMT in a single year. Table 1 lists data pertaining to global plastic production.

Plastic films see use in many aspects of society and have a high global production rate to match their demand. Flexible plastics are reported to constitute approximately 22% of all plastics produced [6,10], becoming waste within a year of their production as single-use plastics [6]. Production and demand for flexible plastics are directly tied to their application in packaging food and liquids due to plastic’s outstanding physical properties, prevention of contamination, and mitigating decay mechanisms in perishables [11,12]. Plastic films are a primary packaging material and have surpassed paper as the dominant packaging material due to superior qualities in plastics [9]. The benefits of flexible plastics to society have had undesirable consequences, including microplastic release in food and marine environments, uncontrolled pollution into ecosystems, and emissions from the production of flexible plastics [13]. When microplastics are ingested or internalized within humans, complications may arise, including cancer, birth defects, and other diseases in human physiology [14]. Plastic waste buildup has become a bigger problem every year, with the United Nations (UN) warning of environmental and ecological problems from plastic waste buildup [15] These dangers require coordinated actions from a global to local level to prevent further flexible plastic waste buildup [13]. Dependence on plastics for everyday use has led to serious environmental and health concerns that require plastic film waste buildup to be addressed.

All plastics produced eventually reach the end of their lifecycle and are discarded, necessitating end-of-life treatments of plastic waste. While policies on the elimination of multilayer films from plastic applications, replacement with other types of materials, or substitution with easier-to-recycle materials are debated to mitigate future issues with plastic waste, recycling is still the major route to divert plastic waste from landfills [16]. The global market value for recycled plastics is estimated at 72 billion USD by 2027 [17] and is an underdeveloped field, as annually, an estimated 80 to 120 billion USD is available in plastic materials that are landfilled [4]. Discrepancies between the projected market values of recycling industries compared to the growth of food packaging materials indicate that there is ample room for recycling to grow. Plastic waste, and where it ends up, is also quantified in order to show the severity of the problem. However, data availability is limited when discussing waste streams and plastic waste composition and content [18]. Reported information should be considered approximations of plastic waste numbers. Table 2 lists data on all plastic produced globally from 1950 to 2015 and contains specific estimates pertaining to end-of-life treatment of plastic waste generated.

Plastic waste composition can be further reduced to the number of flexible plastics in both global and national-level waste streams. Global statistics from Table 1 indicate at least 79 MMT of flexible plastics being produced from single-use plastics alone. Single-use plastics are designed to be discarded after their purpose is served, as seen primarily in food packaging [19]. Approximately 90% of single-use plastics contain polypropylene (PP), high-density polyethylene (HDPE), low-density polyethylene (LDPE), linear low-density polyethylene (LLDPE), and polyethylene terephthalate (PET) [4]. Having plastics be single-use leads to waste buildup, as the lifecycle of these types of plastic is short. After one year, up to 80% of plastic packaging production volume may be considered waste at that point [20]. As of 2022 in the United States, flexible PET plastic films account for approximately 2% of collected PET-based waste plastics, or about 213 kilotons of flexible plastic waste, and are often heavily contaminated, affecting the recycling potential at Material Recovery Facilities (MRFs) [21].

**Figure 1:**
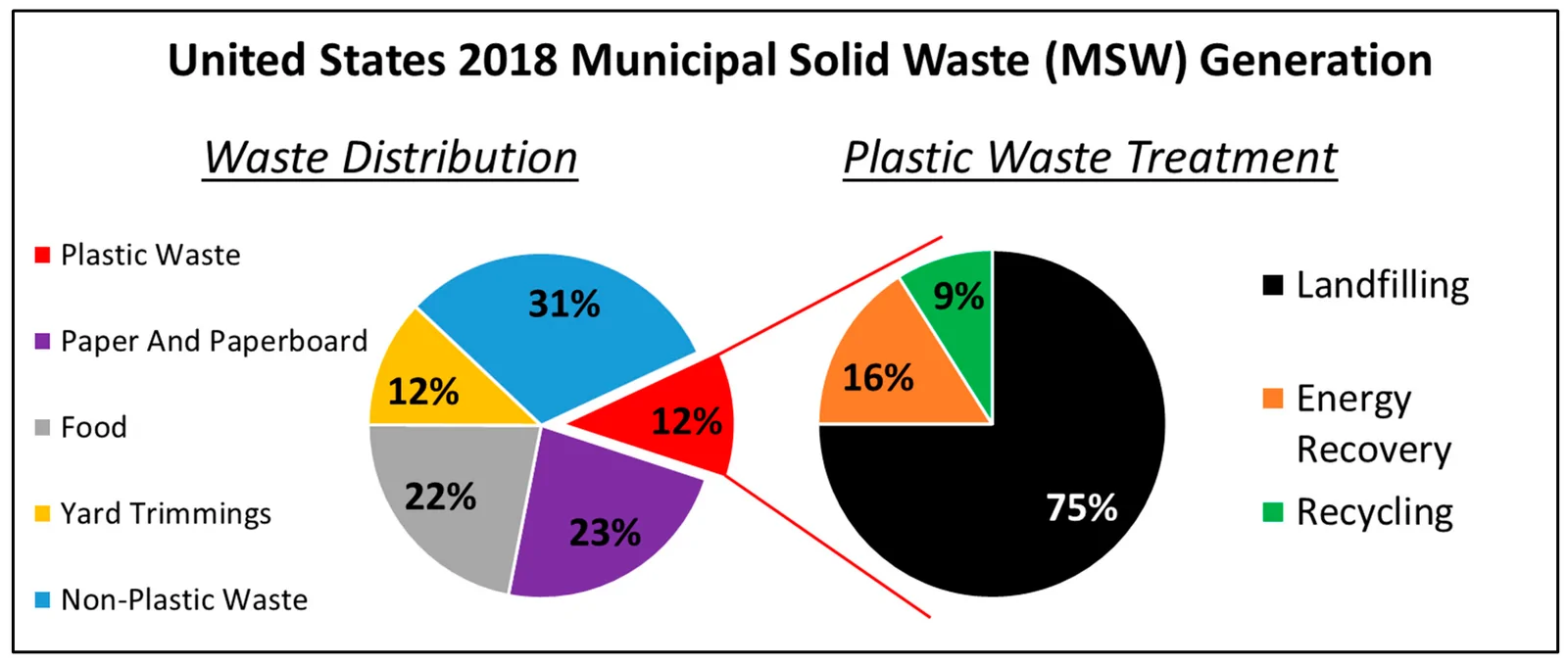
United States municipal waste stream data from 2018 [24].

Municipal solid waste data from the US shows the percentage of plastic seen in waste streams, and the methods of plastic waste treatment on a national level. In 2016, the US was estimated to have generated 42 MMT of plastic waste and was the highest plastic waste producer globally [22]. This waste generation grew to 44 MMT by 2019 [23]. This growth in plastic waste generation indicates the need for proper waste management to minimize problems. Reports from the US Environmental Protection Agency (EPA) indicate that only 9.3% of plastic waste is recycled and 75.4% is landfilled [22]. These numbers reported by the EPA are from 2018 and have not been updated at the national level since then [24]. Figure 1 is a graphical representation of the amount of plastic waste in the US and how it is disposed of.

Another example of plastic waste quantities at a national level is from Austria. While roughly 40% of European plastic production went to packaging, with less than 30% of generated waste being recycled [25], Austrian waste management reported that, in a sample of approximately 170 kilotons of collected waste, 30% was flexible plastic packaging, with further characterization of this plastic waste being done via Fourier-Transformed Infrared Spectroscopy (FTIR) to identify polymer types present [26]. Plastic films comprised up to 56% polyethylene, 51% PP, 14% PET, and 7% polyamides [26].

Plastic film waste management presents a grand challenge in modern times and, as such, has a great amount of research being performed on various aspects of recycling technologies. Several literature reviews of plastic recycling methods have been published in recent years. Horodytska et al. have a great collection of plastic film recycling techniques and developments till 2017, and serve as an excellent starting point for plastic film recycling overviews [6]. More recently, reviews have come out for film recycling in 2022 [27], 2023 [28], 2024 [29,30] and 2025 [31,32] to present novel plastic film recycling techniques. These recent reviews all have a good collection of information but either focus on one specific method of film recycling [30,31] or focus on potential environmental dangers more than the recycling technologies being developed [27,28,29,32]. This motivates an update of current recycling technologies for plastic film.

The goal of this review is to address plastic film recycling specifically, especially processes that can handle a feedstock with a heterogeneous structure, like multilayer films, and highlight emergent efforts to advance plastic film recycling, such as delamination separation technology [33]. This review begins with an introduction to what flexible plastics are and their production, followed by an overview of how plastics recycling is generally done for any and all types of plastics. Established plastic film recycling technologies are then presented, followed by the specifics of novel recycling technologies developed in recent years to address the recycling challenges disclosed. Technologies explored for multilayer plastic film recycling include compatibilization, dissolution–precipitation, delamination, and enzymatic depolymerization for PET films. Highlights of recent developments in plastic film recycling are organized in a table format.

## 2. Flexible Plastic Films

### 2.1. Properties of Flexible Plastic Films

Flexible plastics, or plastic films, are a useful material that is consumed globally. Plastic films are used primarily in packaging applications for consumer products, and also in agricultural or construction materials [1]. Plastic films are classified as either monolayer films, used in agriculture or construction, or multilayer films, which are typically used in food and medicine packaging [1]. These films may contain additives, inks, and dyes to meet consumer specifications, or have printed marketing information on the films [34]. To meet consumer demand and the specifications of a certain application, plastic films are designed around these challenges. As a material, plastic benefits from having properties that can be controlled and engineered to optimize its effectiveness. Thin, flexible films of plastic serve as an excellent choice for a lightweight material for industrial and consumer applications. In packaging applications, plastic films are typically designed to serve as gas or moisture barriers [35]. Polymer selection is based on its properties. For example, ethyl vinyl alcohol (EVOH) serves as an excellent oxygen barrier [35]. Common polymers used in films include PP as a moisture barrier, HDPE, LDPE, or LLDPE for bulk filling, PET for mechanical strength and gas barrier, and polystyrene (PS) for printing and gas barrier applications [4,35]. Polyethylene (PE) polymers like HDPE, LDPE, and LLDPE are also heat-sealable and are good for combining with other polymers to make multilayer films [35]. Polymers that serve to provide mechanical strength in films include poly(vinyl chloride) (PVC) and polyamide (PA) [35]. Depending on consumer needs, different polymers are selected based on their properties to design a film with appropriate properties. Table 3 summarizes the common functions of different polymers and materials utilized in multilayer film compositions.

Polymers are relied upon so heavily for packaging applications, as they have superior properties when compared to alternative materials. Compared to aluminum foil and glass, two competing materials for packaging, polyethylene provides the most flexibility, greatest elongation, and lightest weight, and is the lowest-cost material [37,38,39,40,41,42]. Values for these comparisons can be seen in Table 4.

**Figure 2:**
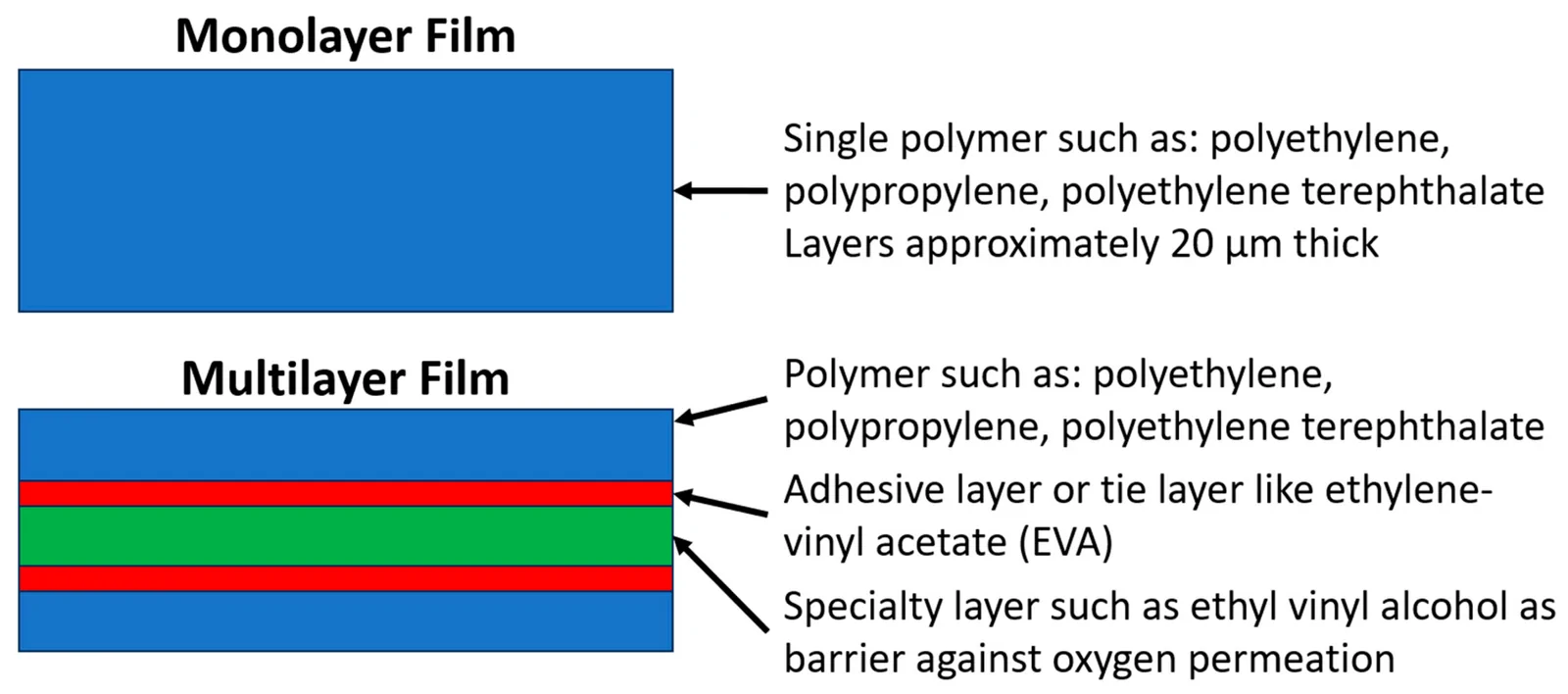
Simplified diagrams of monolayer and multilayer plastic films [6,35,43].

Monolayer films are composed of a single polymer, like polyethylene, and are produced via extrusion for agricultural or construction uses, while multilayer films are produced by coextrusion or lamination and combine a combination of polymers to make advanced barriers that work against gases or moisture [6]. Multilayer film design varies based on application use but follows a general principle. The films have thicknesses between 20 and 400 µm and must be composed of at least two layers [6,34,43]. Multilayer films are designed as a combination of polymer types with the goal of optimizing film properties. For example, if designing a film for use as an oxygen barrier, the sequence of polymers in the film can impact the barrier properties. Having a multilayer film structured as PP/EVOH/PP results in higher oxygen permeability than polycarbonate/EVOH/PP [44]. This interaction allows for greater customization of film properties and promotes optimization of multilayer films. Figure 2 depicts the design of the two main classes of films discussed above.

Multilayer films are designed with an optimal order of polymer layers to maximize film properties. Should two polymer layers not properly adhere together, there needs to be a tie layer to help adhere different layers together [45]. The additives present in plastic may inhibit layer adhesion and binding [45]. Since additives are required to meet consumer specifications, a tie layer is used to properly adhere layers together and mitigate adhesion failure. When using additives and tie layers, the tie layer must not interfere with the additive’s functionality either [45]. There are several options when choosing the right tie layer, including grafted copolymers, maleated polymers, and tie resins [45]. Tie layers meet three main objectives: maintain adhesion while a film is under stress, not interfere with the film’s barrier properties, and not ruin the optical properties of a film [45]. These objectives are important, as they correspond to consumer specifications for flexible film used in packaging.

### 2.2. Production of Flexible Plastic Films

**Figure 3:**
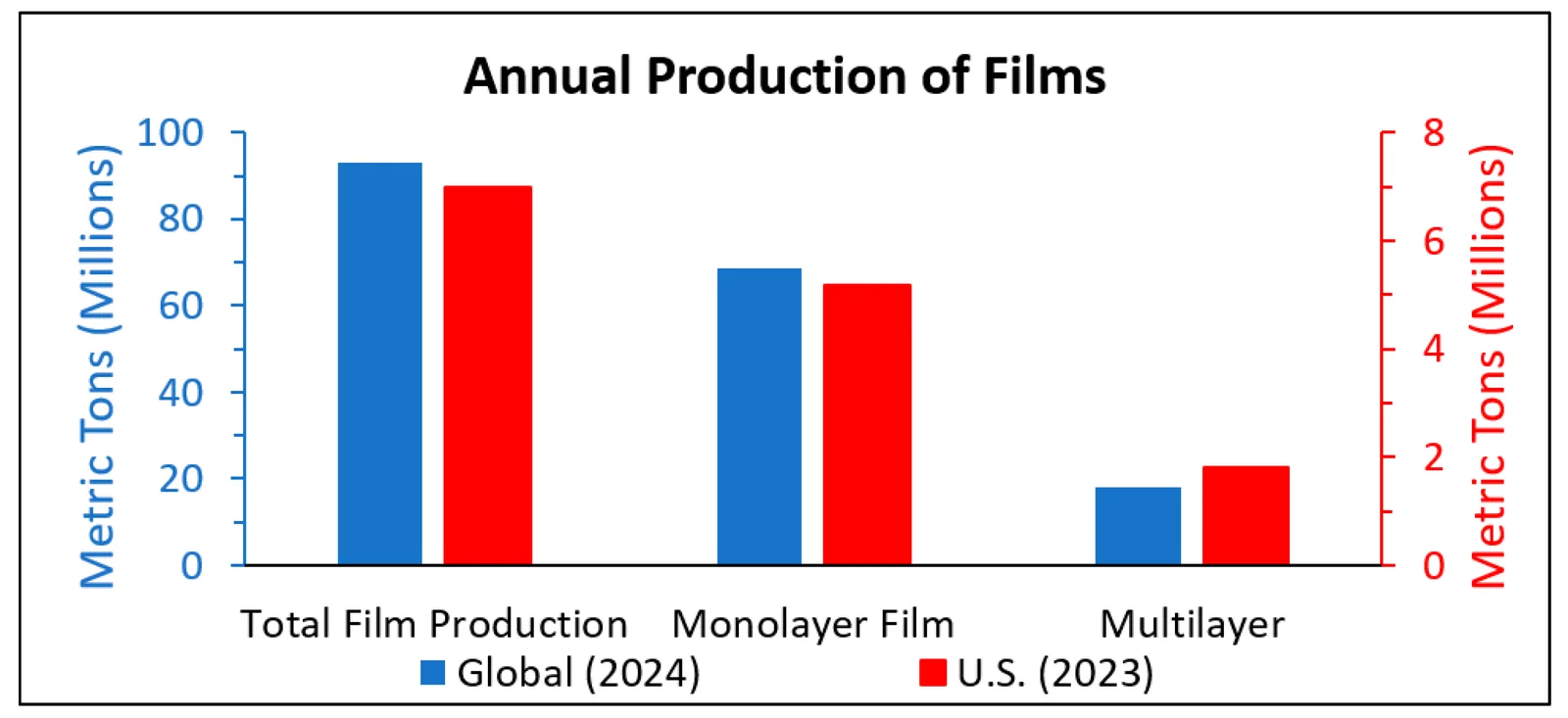
Global and US national annual plastic film production [5,6,8,10,46,47].

Plastic films are a high-demand material, as reflected in their production rate. As of 2023, global production of films amounted to approximately 89.4 million metric tons, constituting about 21.6% of total plastics production for films alone [6,8,10,46], with the US as the largest market for plastic films [47]. Flexible films excel in packaging markets, and the reliance upon them in flexible packaging is seen in the amount produced. Market values for plastic films are expected to grow in the coming years [47]. Film production can further be split into monolayer and multilayer film production. Multilayer film production is estimated to be 26% of total film production [6]. Figure 3 is a graphical representation of the annual production numbers of plastic films from 2024 globally and from 2023 for the U.S. domestically. Reliable information on domestic plastic film production is limited; estimates from 2023 serve as a reference point, with the understanding that these numbers would only increase at a rate comparable to that of total global plastic production reported in Table 1.

The production of plastic films can be conducted on a large scale in order to meet consumer demands. There are different production processes depending on whether the film is to be monolayer or multilayer. Monolayer films can be produced by blown or cast extrusion, while multilayer films can be made by cast extrusion or lamination [48,49,50]. Blown extrusion is a simple process that involves extruding heated polymer that is shaped with a die to make monolayer films [48]. An example setup for blown extrusion can reach 75–150 kg of film produced per hour, with the film being 0.008 mm to 0.1 mm thick [51]. Plastic bags are one of the products that are made from this type of extrusion. Cast extrusion also uses heated extrusion and a die, but may make multilayer films via coextrusion, with multiple polymer layers produced simultaneously [49]. Example production values for cast extrusion include 600 m of film produced per minute or 1000–1100 kg of film per hour, all while being able to coextrude three polymer layers [52]. Lamination works by adhering or laminating film layers together to create a multilayer film. There are two subtypes of lamination: adhesive lamination, which binds layers together with adhesives, and extrusion lamination, which uses molten resin to bind polymer layers together [50]. A three-layer lamination machine can produce up to 1500 m of film per hour, at a precision of 0.1–5 mm thickness [53]. Additives may also be included in a film’s production in order to improve various aspects. Some examples of additives are plasticizers used to bolster the flexibility of plastic films, antioxidants to prevent ultraviolet light degradation, and fillers like talc or calcium carbonate [54]. Additives are important to plastic films. However, there are unintended effects from using certain additives. When additives are released from plastic films, they can become pollutants in water or soil and pose both environmental and physiological risks [54]. All films are designed with specific applications in mind, and the use of additives helps achieve sufficient properties in films. Table 5 lists common additives used in plastic films and their typical composition in a film by mass.

## 3. Current Recycling of Plastics

A general overview of plastics recycling is discussed here to provide background on the field and the application of the core recycling methodologies. This coverage is representative and not comprehensive, as there is a very large literature pertaining to plastics recycling, and it is outside the scope of this review to cover it [55,56,57,58]. Recycling offers a way to recover materials and incorporate them into a circular economy. Environmentally, recycling can reduce energy consumption, CO_2_ emissions, and fossil fuel dependence, as opposed to the production of virgin materials for use in plastic products [59]. Recycling methods need to produce a certain quality of recycled material. If the recycled material is low quality, e.g., has a reduction in mechanical properties, it cannot be substituted into feedstock or replace a product [60]. Due to the complexity of recycling processes and the combinations of waste feedstock and the materials used, general comparisons of economic and environmental impacts are challenging to make and are often not feasible. Laurent et al. [61] analyzed a collection of over 200 life cycle assessment (LCA) studies related to plastics recycling. However, the authors note that the complexity of not only where the waste comes from but also the methods used for logistics, recycling, and reprocessing leads to results that render direct comparisons between studies ill-advised.

**Figure 4:**
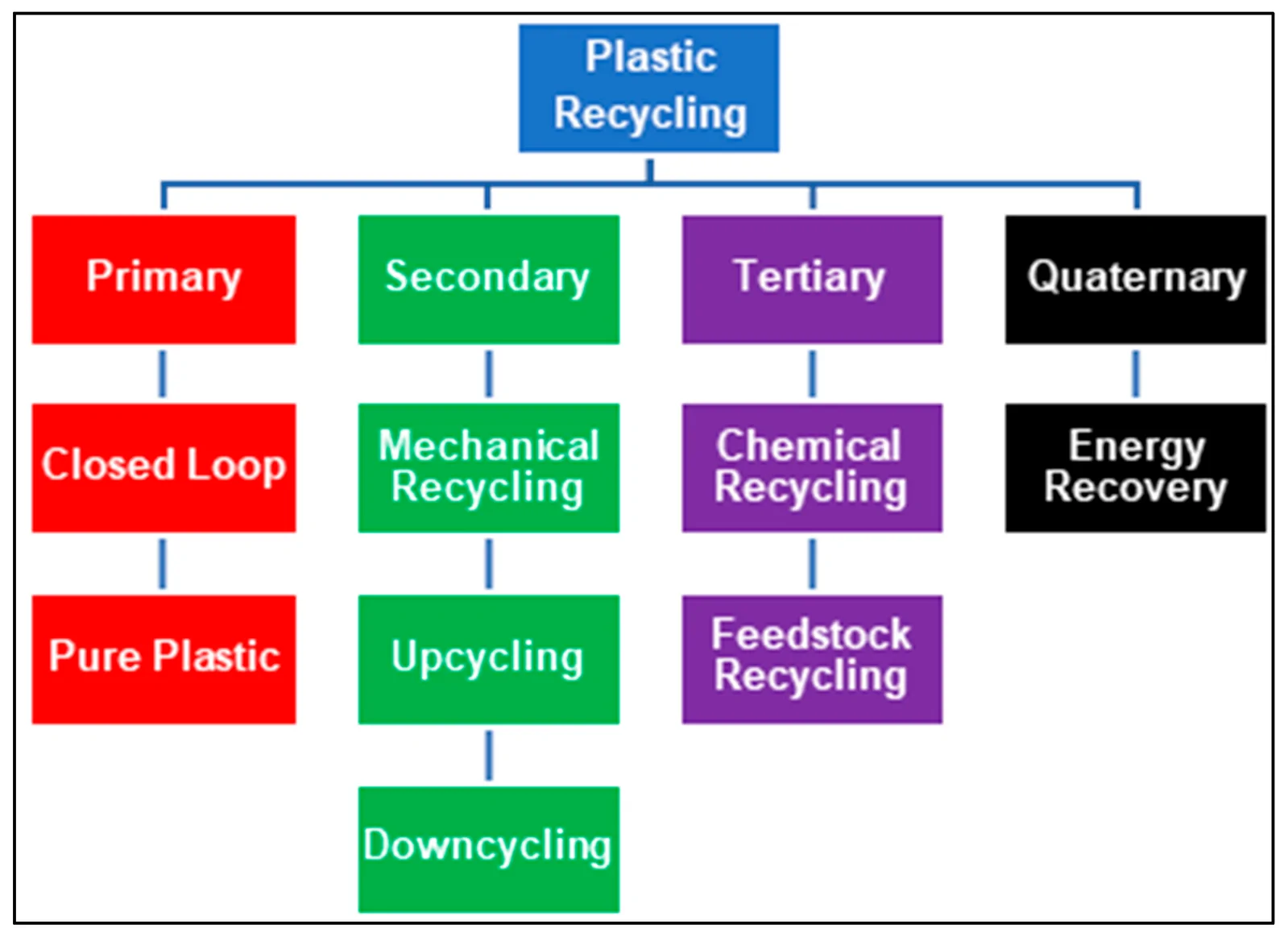
Types of recycling for plastics [63].

Processes for plastics recycling are often classified as primary, secondary, tertiary, and quaternary [62]. Each corresponds to different goals of recycling within each type. Primary recycling is closed-loop and mechanical. Secondary recycling is mechanical recycling and open-loop. Tertiary recycling is chemical recycling, and quaternary is energy recovery [63]. Energy recovery, primarily by incineration, is not typically considered as recycling due to the combustion of plastic into CO_2_ [64]. However, it is still discussed here due to the dependence on incineration as an end-of-life treatment method for plastic waste. Closed-loop recycling benefits from the recycled material being close enough to virgin plastics in terms of properties that the recycled material may act as a direct substitute [65]. Open-loop recycling, like mechanical recycling, results in the recycled materials not being the same quality as virgin plastics, so they can still be reused, but may not replace virgin plastics in the same applications [65]. Mechanical recycling and chemical recycling allow recycled materials to go through upcycling or downcycling. Upcycling involves using additives (e.g., chain extenders) on recovered materials to improve properties to a higher performance, while downcycling is akin to open-loop recycling in that downcycled materials suffer some degree of deterioration [66] and cannot directly substitute for plastic product production [67]. Figure 4 is a diagram representing the four types of recycling and the processes associated with each recycling type.

Within each of the four types of recycling, there are various recycling processes that are developed with targeted polymers in mind. Some recycling methods are better than others in dealing with specific plastics, contamination, and additives. The concept of upcycling or downcycling plastic waste is not limited exclusively to mechanical recycling, but rather is a classification of how recycled material is processed for reuse applications [63]. Table 6 lists common processes in each recycling type, as well as the corresponding polymers targeted as feedstock. Secondary, tertiary, and quaternary recycling are expanded upon in this section in order to provide a better understanding of what the recycling processes are capable of.

### 3.1. Mechanical Recycling

Mechanical recycling follows a process of sorting incoming plastic waste and separating desired plastic types (e.g., HDPE or PET), washing the recovered plastic objects to mitigate contamination, size reduction of the plastic waste, and then reprocessing of the plastic, which typically involves additives or compatibilization and extrusion [89]. Mechanical recycling requires strict sorting of waste streams. Unknown waste stream composition is a major inhibitor of the mechanical recycling process. Sorting collected waste is challenging, as waste comes from a wide variety of sources and all with varying levels of contamination [98]. Flexible plastics compound this difficulty, especially multilayer films, as they need to not only be separated for specialized treatment, but the composition of flexible plastic waste determines what recycling process would be viable [99]. This type of mixed plastic waste makes multilayer flexible plastics resistant or immune to the sorting methods tailored for solid plastics, like sink–float sorting [100].

Characterization is not an impossible task, however, and improvements to sensor technology for plastic waste identification improve the recycling process [101,102]. Use of near-infrared (NIR) or Raman spectroscopy can allow for polymers to be identified based on their spectral signatures [77]. In addition to characterizing the rest of the waste stream, black plastics bring a unique set of challenges with them. When they are placed on a black conveyor belt, black plastics on a black surface cause difficulties with sensor technology [99]. Implementation of artificial intelligence (AI) for sorting is being developed and could serve to improve sorting of plastic film waste [101,103]. Improper sorting or misidentification of plastics prior to mechanical recycling results in severe impairment or total failure of compatibilization techniques, which are heavily reliant on knowing which polymers are in a mixed waste stream [89]. It is not ideal to have to rely heavily on sorting for mass waste treatment, as it limits the amount of plastic waste that can be treated [89].

After the sorting of plastic waste, washing and decontamination can begin [72]. Devolatilization of plastic waste is also performed in order to facilitate recycling via reduction of residual volatile organic compounds (VOC) [104]. Size reduction is the next step and is done by granulation [70], shredding [72], or grinding of the plastic [20]. Extrusion with or without the use of plasticizers [69] or injection molding [89] can then be done. Energy consumption during mechanical recycling, specifically extrusion, has room for improvement during treatment [72]. Mechanical recycling also benefits from being closed-loop or open-loop in design. Closed-loop pathways involve a plastic being recycled into the same product for continual cycles, but is prone to degradation [66,105,106,107], while open-loop takes in additional recycled material [20]. This degradation, resulting from heated extrusion under high shear of the recycled materials, or downcycling, limits the number of times a polymer can be recycled mechanically [105,108].

### 3.2. Chemical Recycling

Chemical recycling offers a complementary approach to mechanical recycling and is useful in treating plastic waste that mechanical recycling cannot process. Mechanical recycling favors rigid thermoplastics, single polymer-type feed streams, and is designed around physical treatment of plastics. Chemical recycling can be used to process plastic waste unsuitable or undesirable for mechanical recycling, such as flexible plastic films, polyolefin blends, thermosets, or mixed waste streams. The end-goal of chemical recycling is to produce a feedstock for future plastics production, but the technology requires further advances to reliably generate products that can be reprocessed into resin that may see usage in applications equivalent to the original plastic product [29,68]. Chemical recycling includes pyrolysis, but also other processes such as gasification for polyolefins, depolymerization for PET, and solvent-based processes that do not break polymer chemical bonds, such as dissolution–precipitation.

Pyrolysis is the most common chemical recycling process and involves high temperatures to turn waste plastic into pyrolysis oils and waxes for feedstock or energy uses [84,87,109,110,111]. Two important variables in pyrolysis are temperature, ranging from 300 °C to 700 °C, and reactor design for the process [95]. Non-catalytic pyrolysis requires even higher temperatures, needing a range of 350 °C to 900 °C depending on the feedstock from plastic waste [112]. Feedstock streams can be treated before pyrolysis to optimize the process. Pyrolysis offers an option for recycling multilayer plastic film [84]. Post-consumer waste, often rich in contaminants, may be processed via pyrolysis, despite high contamination of the feedstock [85]. Contaminants can be destroyed during pyrolysis. Solvents based on paraffin wax can be mixed with polyolefin waste to lower viscosity and promote pyrolysis toward liquid products via liquefaction [113]. In a similar vein to pyrolysis, hydrocracking involves turning long-chain polymers into shorter chains, primarily for the production of fuel [68] via the removal of hydrogen and the use of a catalyst [114]. High pressures, around 70 atm, 400 °C, and the presence of a catalyst are examples of conditions used during the hydrocracking process of plastic waste [89].

There is also gasification, a method to produce hydrogen for fuel use [92] and CO from waste plastic, using temperatures of 900 °C or greater [115], to this end. Breaking down polymers, however, is not without challenges. Gasification relies on inputs of heat or steam to produce hydrogen, but also has carbon emissions resulting from the process [108]. Gasification acts as competition to pyrolysis, using the same feedstock types with thermal treatment [94,112]. As the process efficiency is improving, large-scale industrial implementation is getting close for plastic gasification technologies [68].

Depolymerization, also referred to as solvolysis, is another option for chemical recycling that focuses on converting a condensation-type polymer to its monomers, and then recovering and purifying the monomers to be used for polymerization into the virgin polymer [80]. Depolymerization is applicable for PET-containing waste that contains high levels of contaminants, [76] is designed around PET specifically in most cases [77,79,116,117,118], and is not viable for polyolefins, which are the majority of plastics being produced. Glycolysis of PET would be an example of solvolysis [80]. This method works for multilayer film compositions containing polyethylene and can deal with mixed plastic waste streams since, if the process is designed correctly, only the targeted polymer may be affected [75,76].

Solvent-based recycling is capable of processing plastic film waste as a feedstock source. Dissolution of polymers through solvents is one method of chemically treating plastic waste that gives a product of polymer since it is a physical process. Examples of dissolution–precipitation methods include those for nylon [83], agricultural films [119], and post-industrial film waste [120]. Solubility of polymers and their respective affinity with solvents can be determined with Hildebrand solubility parameters, Hansen solubility parameters, or Flory–Huggins solubility parameters [74]. Solubility parameter values are generally available and allow for an initial approximation of what would be a good solvent for a polymer type, but further experimental and/or computational work is required to define conditions suitable for polymer dissolution and precipitation. The dissolution process would use appropriate solvents at temperatures in the range 70–140 °C to create a stream of dissolved polymer and solvent, from which any undissolved material is separated or filtered out. The resulting polymer solution is treated so that the polymer precipitates with no chemical reaction involved [74].

### 3.3. Energy Recovery

Energy recovery does not constitute recycling, in the sense of recovering a comparable material for reuse in the same application, but can be used if other options for treating plastic waste are exhausted. The goal of energy recovery is designed to turn waste into heat by combustion [94]. Commercial polymers are typically hydrocarbons, which makes them suitable as fuel. Energy recovery exists as an alternative to landfilling plastic waste [121,122,123]. Recent studies into the pathways of plastic waste and benefits of energy recovery as an alternative to landfilling [122] and the economic impact of plastic waste processing methods [121] show that energy recovery can have an important place in plastic waste management. The major hindrance to energy recovery is that the plastic waste being processed this way is not directly being recycled into an equivalent product, leading to a continued need for virgin resins and fossil oil dependence.

## 4. Recycling of Plastic Films: Established Technologies

Plastic films are a challenge when it comes to recycling. Attempting to eliminate the use of, reuse, or the substitution of, plastics are good steps to reduce overall film waste volume, but are insufficient and/or not practical [16]. Plastic film waste treatment may be approximated as follows. Landfilling/disposal accounts for an estimated 50%. Mechanical recycling is near 25%, energy recovery constitutes around 24%, and the remainder is processed via chemical recycling [6]. The design of plastic films creates difficulty in finding a recycling process that works. For example, food packaging is designed to preserve a product and prevent damage, and having a plastic film resistant to degradation is important [124]. Functionality as gas barriers, moisture barriers, and permeation resistance inhibits existing recycling methods from integrating plastic films. Because flexible plastic is often used in a consumer environment instead of an industrial one, contamination also restricts recycling viability. High contamination concentrations from post-consumer use or additives, inks, polymer choices of plastic films, and the low bulk density of films are all challenges when looking to recycle films. Multilayer plastic films are a particularly challenging feedstock to use, given their combination of bound polymers with adhesives, tie layers, additives, and potential non-intentionally added substances acquired during their life cycle [125,126,127]. A key issue when recycling films that were used in primary packaging applications, specifically for food and medicine, is that they are downcycled to non-packaging products due to health and safety concerns [128].

Another drawback of recycling plastic films, and recycling in general, is a lack of understanding of sorting post-consumer waste. Post-consumer waste is often poorly sorted due to limitations. For example, it is not expected that a consumer would sort a monolayer film from a multilayer film [26] or that municipalities even collect films for recycling [62].

The recycling of plastic film waste is needed, as films are too effective at their applications, and substitution and elimination of these materials would not be reasonable [16]. Therefore, improvements to recycling technology must facilitate such recycling of film. Four different recycling processes are leading the movement to include plastic film waste in recycling: mechanical recycling, pyrolysis, dissolution–precipitation, and delamination. Mechanical recycling is a mature technology that is constantly advancing to incorporate additional types of plastic waste to use as feedstock, while pyrolysis has been a focus of research for nearly half a century and has plastic film waste as a potential feedstock. These technologies are established in the recycling space, and the inclusion of film waste as feedstock for these technologies is critical to addressing plastic film waste treatment.

### 4.1. Mechanical Recycling of Plastic Films

When looking at plastic films in mechanical recycling, there are obstructions that prevent plastic films from being recycled mechanically. Limitations of mechanical recycling to treat flexible plastics have resulted in sorting systems separating out plastic films, and limited film feedstock from post-consumer sources [62]. Furthermore, films are usually exposed to contaminants during their life cycles and also have many additives, colorings, or fillers that make mechanical recycling difficult or impossible [126]. Mechanical recycling also relies on size reduction of incoming plastic waste for reprocessing steps like extrusion or molding. Flexible plastic films in mechanical recycling feeds tend to entangle the equipment and clog the process [84]. Another problem with films is specific to multilayer films. Polymer sorting is required for mechanical recycling, as it improves the quality of recycled materials. But, multilayer films contain difficult-to-separate polymer layers and would not be viable for this type of recycling [69]. Multilayer films, being a mix of different polymer types based on the layers present, are not able to be recycled mechanically. Challenges surrounding plastic films make mechanical recycling an unlikely candidate to resolve the buildup of flexible plastic waste. Monolayer films stand a better chance of being recycled mechanically, but are still problematic, while multilayer films need careful design. MRFs are not inclined to take plastic film waste, as it would not be a viable resource for collection.

To address these issues, there have been improvements to the mechanical recycling of plastic films. Waste Management has disclosed a pilot scheme for recovering plastic film waste in an Illinois city as of 2022 [129]. This pilot would cover 3500 houses and recover an estimated 120,000 metric tons of plastic film waste annually [129]. Recovering the plastic film waste and preventing it from going to landfills is a better alternative. Improvements to the sorting and identification systems used in mechanical recycling MRFs are another improvement to plastic film recycling. AMP Robotics has developed an AI-powered sorting system to properly sort films that is designed to be retrofitted onto existing MRFs that lack the capabilities to recycle plastic films [130]. Sorted films could then be baled and transported or sold to groups that can recycle the waste. Designing mechanical recycling equipment to process film waste is another recent advancement. EREMA is a company that produces extruders capable of handling both post-industrial and post-consumer plastic film waste for mechanical recycling [131]. Damage to extruders is a major inhibitor to mechanically recycling plastic films. Designing an extruder capable of handling film waste as feed nullifies this concern. Should advancements in mechanical recycling continue, in order to address challenges with mixed plastic waste [132], contamination [133,134], and multilayer plastic film structures [135], plastic films will eventually see consistent use as viable feedstock for mechanical recycling MRFs [136,137]. In 2026, a report of an advanced mechanical recycling facility seeks to process 50,000 metric tons of flexible plastic waste per year and produce nearly 25,000 metric tons of recyclate annually [138]. This pilot plant would also be economically competitive based on assumed pricing from 2023 recycled resin values [138]. This pilot MRF would be a primary example of implementation of new mechanical recycling technologies to process flexible plastic film waste.

### 4.2. Pyrolysis of Plastic Films

When mechanical recycling is not a viable option for plastic films, chemical recycling is an alternative option, with pyrolysis being explored for treating plastic film waste. Pyrolysis of plastic films involves thermal decomposition of long-chain polymers, primarily polyolefins, at high temperatures and no oxygen [139,140,141,142]. For pyrolysis, the liquid product, pyrolysis oil, is desirable for use as fuel or plastic production [109,139]. Concerns regarding pyrolysis include the process’ energy consumption, low-quality products, and the environmental impact of fuels made from pyrolysis oil. Most pyrolysis reactors run at a minimum of 500 °C in order to decompose mixed plastic film waste [139,143,144]. Despite high temperatures, waxes and paraffins are often incomplete products generated during pyrolysis and need further processing to be turned into a liquid product of desired specifications [109,139]. Pyrolysis can handle a feedstock of mixed plastic film waste and divert it from the landfill or incinerator, but the production of pyrolysis oil and its environmental impact are considerable concerns. Burning diesel as a fuel gives off carbon emissions, and the use of naphtha to produce more plastics could be seen as perpetuating environmental problems. These concerns are considered when planning to recycle plastic film waste, especially when options are limited.

Pyrolysis does have benefits to it, despite questions regarding end products and emissions. Pyrolysis can handle mixed plastic waste films as feedstock and still produce quality products, including contaminated plastic feedstocks that inhibit mechanical recycling [84,110,111,145,146]. This can be seen with existing pyrolysis plants that rely on flexible plastic feedstocks. TotalEnergies has pyrolysis plants in Spain, France, and the US that can use flexible plastics as feedstock [147,148]. Products from TotalEnergies pyrolysis plants are intended to produce high-quality materials, with a portion to be used for food-grade resins, something that is not commonly done with plastic film waste and promotes the circularity of plastic films [147]. A newly announced pyrolysis plant for TotalEnergies is set to be built in Texas and process “…1 million tons of circular polymers by 2030” and may handle a plastic film waste volume of 310,000 tons per year [147]. Chemical recycling of plastic film waste via pyrolysis provides an opportunity to turn overlooked plastic film waste into a valuable product. Agricultural plastic film has been a recent focus as a feedstock for pyrolysis, including PE film within a biomass mixture, [109,111,146] and mulch films with biogas residue [110]. Multilayer plastic films, or film waste feedstock with multiple polymers present, have also been increasingly used in pyrolysis feedstock, including metallized films [145], in the presence of biomass [142], and thermal characterization and processing [149]. Offering an alternative to mechanical recycling is important. However, the amounts of emissions involved and the high energy consumption with pyrolysis can be problematic and may restrict how widespread pyrolysis of plastic film waste becomes. Improvements to the pyrolysis process to help reduce emissions and energy consumption are imperative to future endeavors with this technology. Of course, the economics of pyrolysis will continue to be affected by the petroleum price, as the products of pyrolysis compete in the market with products from fossil fuel processing.

## 5. Recycling of Plastic Films: Advancements

Motivated by the need for new recycling methods for flexible plastic films, several new technologies are being developed, aiming to address this challenge. Improvements to sensor technology for waste streams can help direct flexible plastic waste to proper recycling and avoid obstructing mechanical recycling. Compatibilization focuses on turning immiscible polymers into a blend through the addition of appropriate additives (Section 5.1). Dissolution involves selecting an appropriate solvent to dissolve a target polymer (Section 5.2). Delamination promotes the separation of plastic film layers for the recovery of different film polymers (Section 5.3). Depolymerization of PET in films to its monomers through the use of an enzyme is another new development (Section 5.4). These methods are discussed in greater detail below. Example cases and a summary of published work are presented in Section 5.5.

Submissions to the Alliance Prize Solutions contest, sponsored by the collaborative R&D agency Advanced Technology International, highlight diverse novel processes that are designed for recycling plastic films [150]. The winner of the submissions was a process using supercritical CO_2_ to wash post-consumer polyolefin films, allowing them to be reused as food-grade materials [150]. Other notable top 10 winners of the submissions include improved separation of multilayer and monolayer waste film by baffled oscillation, a solvent-based washing system for films to promote recycling and remove contaminants, and a full solvent-targeted dissolution process that can recycle the solvent at the end of the process [150]. These examples are important, as they demonstrate recent innovations and technology in plastic film recycling that can potentially be implemented at large-scale.

Despite the promise they hold, the emergent technologies for recycling plastic films that are presented in this chapter are not yet deployed at large scale. Because of this, this review does not incorporate techno-economic components due to a lack of viable data for newer technologies and inconsistent comparison metrics.

### 5.1. Compatibilization

Handling of a waste stream involves the use of characterization and sorting techniques, but these may be bypassed when using a select compatibilizer for a specific mixed plastic waste composition [151,152,153], reducing the overall need to sort and separate polymers [154]. Use of nanofillers and compatibilizers allows for the miscibility of polymers, as well as improvements to mechanical properties like elongation and Young’s modulus [27]. Certain polymer blends are not feasibly recyclable due to the immiscibility of the polymers present. However, compatibilization can be used for select mixed plastic waste that is considered immiscible and provides opportunities for the recycling of flexible plastic waste like multilayer packaging [27].

Films with PE are a good example of compatibilization candidates. In one example, the grafting of PE with maleic anhydride allowed for the compatibilization of LDPE [155]. After being treated with maleic anhydride, LDPE was able to be blended with PA 6 [155]. Modification of the PE film with maleic anhydride provides new recycling opportunities for PE waste films. Additionally, reactive extrusion was done to process the materials following compatibilization [155]. Demonstrating the feasibility of this type of compatibilization and reprocessing could open the way to recycling LDPE-based plastic films and repurposing their materials.

Another example of the potential of compatibilization is that of PLA and thermoplastic starch, as they do not mix until the introduction of a compatibilizer during a molding process [156]. The objective of this compatibilized blend was to be used as a flexible film for food packaging. Therefore, it is important that the film has appropriate properties [156]. Controlling the compatibilization process is centered around finding the right ratio of polymers to compatibilizer, which leads to different effects on the mechanical and barrier properties [156]. Compatibilization allowed two immiscible materials to blend and provided a viable flexible plastic film for packaging use.

Compatibilization offers a method of treating mixed waste for recycling purposes but is also in need of better understanding. Use of two-photon microscopy provided insights into the interactions of a sextuple hydrogen-bonding compatibilizer, ODIN, and a PE/EVOH polymer blend [157]. The compatibilizer used in that work is interesting for several reasons. The hydrogen-bonding potential of ODIN should promote compatibilization, and ODIN is also fluorescent, which allows for better imaging of the interaction the compatibilizer has with the polymer blend [157]. PE was also treated with 2-hydroxyethyl methacrylate to promote compatibilization through the extra hydrogen bonds available [157]. Using a compatibilizer that doubles as a fluorescent molecule is an interesting proposition that could help advance knowledge of polymer blend interactions. That work did show even distribution of ODIN through the polymer bulk and the surface, and that repeated reprocessing could partially reverse the compatibilization and, theoretically, serve as a separating method [157]. Improving the understanding of compatibilization can allow for increased applications and better treatment processes for recycled materials. Further work can be done to see if ODIN can extend to other polymer blends outside of PE and EVOH.

### 5.2. Dissolution

**Figure 5:**
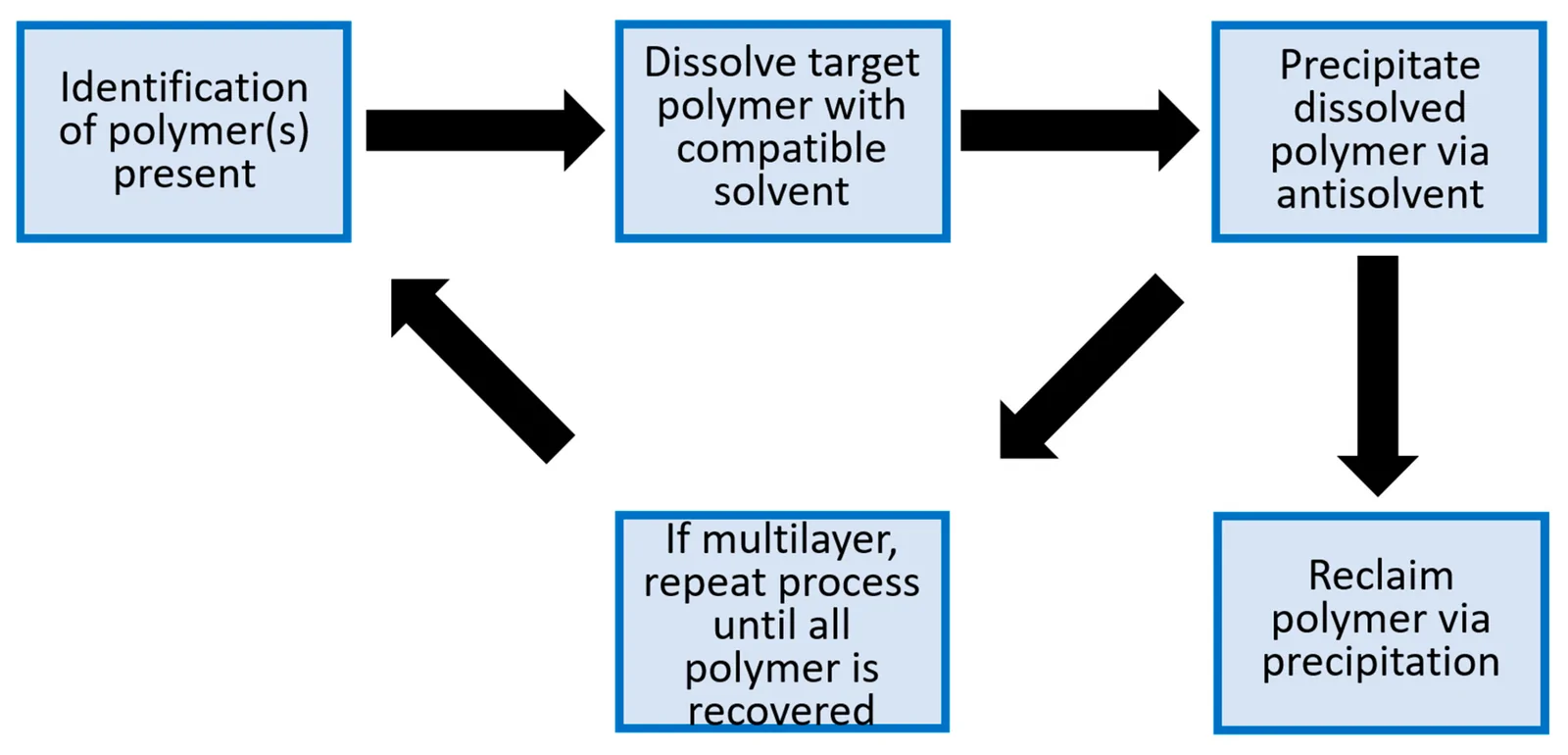
Simplified diagram of solvent-targeted dissolution process [165].

Dissolution–precipitation recycling provides a means of recycling plastic film waste without the high energy consumption and emissions of pyrolysis and is considered another alternative to mechanical recycling [119,158,159,160,161]. This type of polymer recovery excels in dealing with multilayer films, as it can deal with their heterogeneous composition, such as mixtures of PET, PP, and PE [162]. By dissolving targeted polymer layers at elevated temperatures, multilayer plastic films can have their bound layers separated with minimal contamination or degradation, hence promoting material recovery [120,127,163,164]. Figure 5 depicts in a simplified form the process steps needed to perform this type of recovery. Following the identification of the polymers present, an appropriate solvent may be selected to dissolve a targeted polymer. Once that polymer is dissolved, any undissolved material is removed, the polymer solution is precipitated, and the target polymer is recovered for subsequent processing. For the polymers remaining as solids, repeat this process until all of the different polymers are recovered individually, one after the other. This process requires a deep understanding of solvent selection for the polymers involved, and also knowledge of the proper operating conditions needed to quickly induce dissolution while mitigating the risk of complications such as hydrolysis for PET present in films.

Better understanding of appropriate solvent selection is important to further development of solvent-targeted dissolution for plastic film recovery. Use of Hansen solubility parameters or computational modeling [166,167,168] is common to determine solvent affinity, but it is not a perfect method due to being empirical [169]. Implementation of computational modeling could be an option to calculate solvent affinity instead. Using a database of 524 solvents and molecular models of polymers as short-chain oligomers, predictions for solvents using molecular dynamics modeling have been performed [169]. This new technique is beneficial for solvent-targeted dissolution, where the solvent needs to have good affinity for the solute polymer and consideration for whether the solvent may be regenerated for reuse is made [163]. Molecular dynamics (MD) modeling overcomes the oversimplifications seen in Hansen solubility parameters and can also extend to testing new solvents without the need for experimentation [169]. Experimental testing showed that the model was successful for PE and EVOH [169].

While the solvent used to dissolve the target polymer is primarily selected based on solvent affinity solubility parameters such as Hansen solubility parameters, consideration for the type of solvent and its environmental impact should not be ignored. The different solvent types used in dissolution recycling would be organic solvents, green solvents, bio-derived solvents, switchable solubility solvents, and amine-free solvents [163,170,171,172,173,174]. Choosing the appropriate solvent for the targeted polymer enables dissolution, meaning that available data on a solvent is important [175], especially when considering the hazards associated with the solvent [176]. Some solvents may present environmental concerns like aquatic toxicity, acute toxicity, organ toxicity, and other hazards [171]. Green solvents are better from a health perspective, but may be lacking in solubility affinity for polymers and their dissolution [177].

Once the target polymer is dissolved, the non-dissolved materials are filtered out of the dissolved slurry. Precipitation of the target polymer from its solution is generally performed in one of three ways: adding an antisolvent, cooling, or a combination of cooling and antisolvents [59]. The antisolvent added to the system to precipitate the dissolved polymer for collection can then be recovered through distillation [178]. Recovery methods following precipitation typically involve filtration to separate materials [163]. Using antisolvents to precipitate solid residues is an energy-intensive process, since it requires subsequent separation of the solvent–antisolvent mixture, and has considerable emissions. This has led to a focus on cooling-driven precipitation, which involves less energy and fewer emissions [59].

When dealing with monolayer films, this dissolution–precipitation process is simplified, in that only one target polymer is present. Multilayer films are more complex due to their heterogeneous polymer composition. Because of this, a multilayer film can go through several repetitions of this process to recover the recyclable polymers within the multilayer structure [179]. Solvent-targeted recovery and precipitation, or STRAP, is another term for the dissolution–precipitation process and works by taking a characterized and identified multilayer plastic film and using specific solvents and antisolvents to dissolve all polymers present layer by layer [179]. STRAP can be very energy intensive due to the layer-by-layer dissolution and precipitation process and may induce solvent contamination on recovered polymers [59,180]. If the solvent is still present in the recycled polymer’s matrix at the end of the process, the recycled polymer has reduced purity and potentially worse mechanical properties [178]. Recycling via dissolution has certain advantages over other recycling methods, including additive removal and external contaminants accrued during the plastic’s life cycle [163]. Contamination in post-consumer waste is problematic, and solvent-based recycling presents a solution to that.

Dissolution–precipitation for recycling multilayer film waste has been increasingly investigated in recent years. The films used were a mix of PE and aluminum, with the following solvents tested: toluene, acetone, acetic acid, dimethyl formamide, ethyl acetate, and ethanol [162]. Criteria based on safety and environmental concerns regarding the solvent choice were included [162]. Recycling solvents was achieved through the use of distillation in order to recover the solvent for reuse [162]. Over the course of 10 min and at a range of 60–90 °C, there was successful recovery of the aluminum within the multilayer film and partial recovery of the polymer present [162]. This work showed the separation of layers through targeted polymer dissolution in order to recover useful materials from plastic waste.

Post-consumer food packaging with multilayer films that include paper layers can also be treated using dissolution–precipitation. The recycling of Tetra Pak^®^ (Lausanne, Switzerland) involved treatment of a six-layer waste plastic containing paperboard, LDPE, and aluminum, with the paperboard constituting 75 wt% [181]. This type of mixed waste is post-consumer and highlights the advantages of targeted dissolution. Normally, this type of waste would undergo pyrolysis or gasification [181]. Treatment of the post-consumer waste was designed around the different components. Paperboard was first targeted with hydro pulping and then filtered to remove the polymer and metal. LDPE layers were dissolved with xylene and precipitated with isopropanol [181]. The results of this work showed high recovery of the materials present, with only paperboard suffering any losses [181]. With solvent-targeted dissolution, energy recovery was avoided, and the materials could be recovered and integrated into the circular economy.

Dissolution–precipitation recycling has seen advancements as well, despite being a relatively new recycling process. The use of switchable hydrophilicity solvents (SHS), for example, bypasses the need for antisolvents to precipitate solid polymer residue [163,182]. Using cooling to precipitate materials, rather than antisolvents, cuts down on emissions and energy consumption. Dissolution–precipitation recycling has also been considered as a washing treatment for mechanical recycling operations, since dissolution–precipitation can remove additives, contaminants, and separate multilayer films [163]. Recovery of polyolefins using STRAP has also shown good results in recovering resins. STRAP-recovered PE and PET were both close enough to virgin PE and PET resins that they would be considered safe for food use by U.S. FDA standards [46]. A pilot plant at Green Bay, Wisconsin can recycle 500 kg of film waste per hour using STRAP [150]. Dissolution–precipitation recycling can process heavily contaminated plastic multilayer film waste, as solvents are capable of removing contaminants. Using dissolution–precipitation recycling independently or as a pretreatment step for mechanical recycling provides options for its implementation.

While solvent-based recycling technologies hold promise, as they recover intact polymer chains with their full embodied energy, environmental and economic challenges associated with solvent utilization require deeper evaluation. Issues such as solvent recovery efficiency, solvent toxicity, energy requirements, and industrial process integration should be considered in future work in this area. The delamination processing discussed next is a step in the direction of reducing the high solvent consumption associated with dissolution–precipitation recycling of multilayer films.

### 5.3. Delamination

Flexible plastic sees heavy use in packaging, which often includes more than just plastic. The addition of plastic film onto a solid plastic, paperboard, or metal like aluminum leads to waste that is difficult to recycle due to being heterogeneous in composition [183]. Delamination relies on the separation of bound layers in a multilayer film to recover individual single-type materials, generally by utilizing a solvent to dissolve adhesive binding layers in a multilayer film [6,184,185,186] or dissolving a metallic layer that the plastic is bound to, such as aluminum [183]. Once the multilayer film is deconstructed via delamination, the remaining strips can then be separated as monolayer film strips, expanding the possible recycling methods available. Recovery of plastic layers without the polymers suffering any detectable degradation promotes the use of delamination for dealing with flexible plastic waste [187].

Compared to dissolution–precipitation recycling, delamination targets only a small component of a multilayer film and is thus less energy- and emissions-intensive. Furthermore, delamination does not need to go through a multilayer film, dissolving one layer at a time in order to separate the different layers in a multilayer film. One of the main drawbacks of delamination is solvent selection, as there is no standardization for adhesive usage. This can make picking a proper solvent difficult, as the adhesive or tie layer can be composed of many different materials [45]. Delamination can further serve as a pretreatment stage to induce the separation in multilayer films without needing the amount of energy that dissolution–precipitation recycling does.

**Figure 6:**
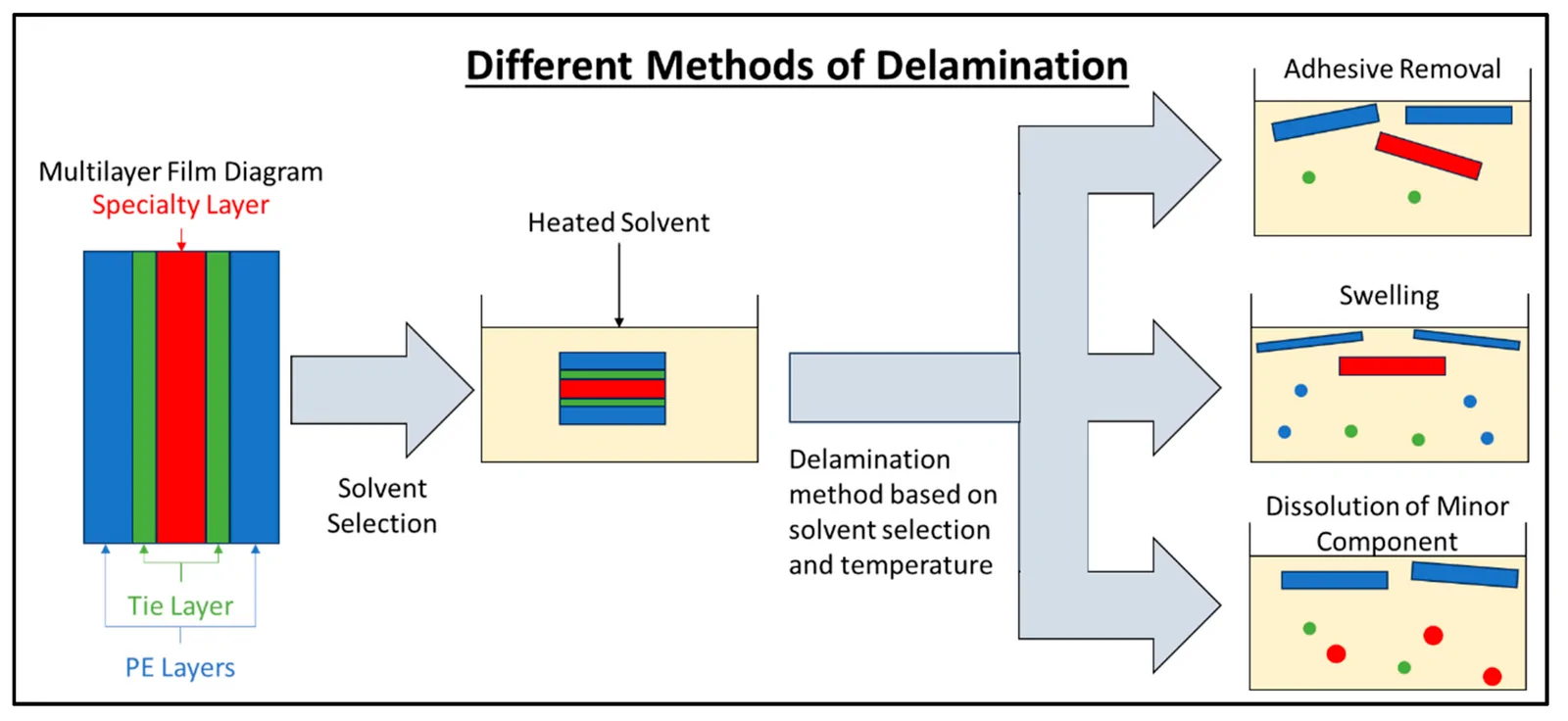
Pathways of delamination [188]. To induce separation, adhesive removal dissolves the adhesive tie layer, swelling targets a polymer to partially dissolve and swell, and finally a minority by mass component may be dissolved to separate bound materials [33,189].

Figure 6 depicts three pathways of inducing delamination, namely adhesive removal, swelling of a polymer layer, and dissolution of a minority-by-mass layer. Adhesive removal involves the dissolution or degradation of the adhesive to unbind all polymer layers for recovery. In swelling, the solvent targets a specific material to be swollen and unbind as a solid film, freeing up other materials as a solid film. In dissolution of a minor (by mass) component, the solvent selectively dissolves a component of the film, such as Nylon 6 or EVOH, to unbind, as solid film, the majority (by mass) polymer targeted for recovery.

Delamination may be induced in different ways depending on the multilayer film feedstock used. For non-metallized films, targeting the adhesive layer with an acid, such as formic acid, can be successful in separating polymer layers as solid film. Films containing PET may be swollen with m-cresol to delaminate solid PE and PET layers. For feedstock that is resistant to the other pathways, targeting a larger specialty layer like EVOH or Nylon 6 can induce delamination via targeted dissolution. Delamination is able to be induced in under 1 h and at temperatures at or below 80 °C [189].

Delamination relies on targeting a specific layer of the flexible plastic waste to make the waste recoverable. One method is through selective glycolysis of the adhesive within the plastic waste [165]. Removal of an adhesive layer would promote separation and allow for recovery of the different layers present in the flexible plastic waste. PU was the target adhesive in this example, and diols were used to treat PU under mild conditions [165]. Further, the solvent selection criteria went beyond affinity with PU. Work was also done to show solvent recovery and carbon emission modeling regarding the solvent selection [165]. This work shows the selectivity of targeting PU adhesive and the ability to separate flexible polymer layers from each other under the proper conditions.

For recycling to be effective in dealing with waste buildup, the criteria should extend to the effects of the process itself. Carboxylic acids can act as good delaminating agents in PET/PA and PET/PE films, but the environmental impact that they have may be a hindrance on a larger scale [187]. Kinetic modeling of a known delamination process provided greater understanding of the delamination mechanisms and solvent pathways involved [187]. The generated kinetics data allowed for the optimization of the delamination process, while also considering carbon emissions and energy consumption, in order to improve the process in more than just a reduction of delamination time [187].

The permeation of the solvent used in separating plastic from other materials can be a limiting factor for the delamination process. Treatment of the plastic waste by microperforation could promote permeation and the delamination process [190]. Perforation of a multilayer flexible package resulted in greatly decreased delamination times, and the increase in solvent temperature also caused a reduction in delamination time [190]. The polymer used, PET, was delaminated from a metal backing of aluminum by dissolving the aluminum with caustic soda [190]. This type of pretreatment could help on an industrial scale as well. Since perforation exists in mechanical recycling, the pre-treatment of flexible plastic packaging by microperforation could lead to reduced treatment times on a larger scale [190]. Focusing on the improvements to solvent transport would very likely result in improvements to the delamination treatment method, primarily via reducing the time needed to process films.

Recent work has seen the successful delamination of multilayer plastic film with a focus on PE recovery without the presence of a metallized layer [33]. Utilization of formic acid as a solvent to dissolve PU-based adhesives has led to the timely separation and recovery of PE as solid film [33]. The delaminating solvent used can be recovered and recycled via distillation to promote a closed-loop recycling process that mitigates material consumption [33]. The mechanical properties of the recovered and recycled PE were found to be comparable to commercially available PE resins, indicating that the delamination process could recover polymers of comparable mechanical properties, a key step needed to show that polymers recycled via delamination may eventually be used in an equal application to the original virgin resin [33]. Delamination’s applications also extend to post-consumer film waste, showing application to metallized salted snack packaging and confectionery packaging.

### 5.4. Enzymatic Depolymerization of PET Films

**Figure 7:**
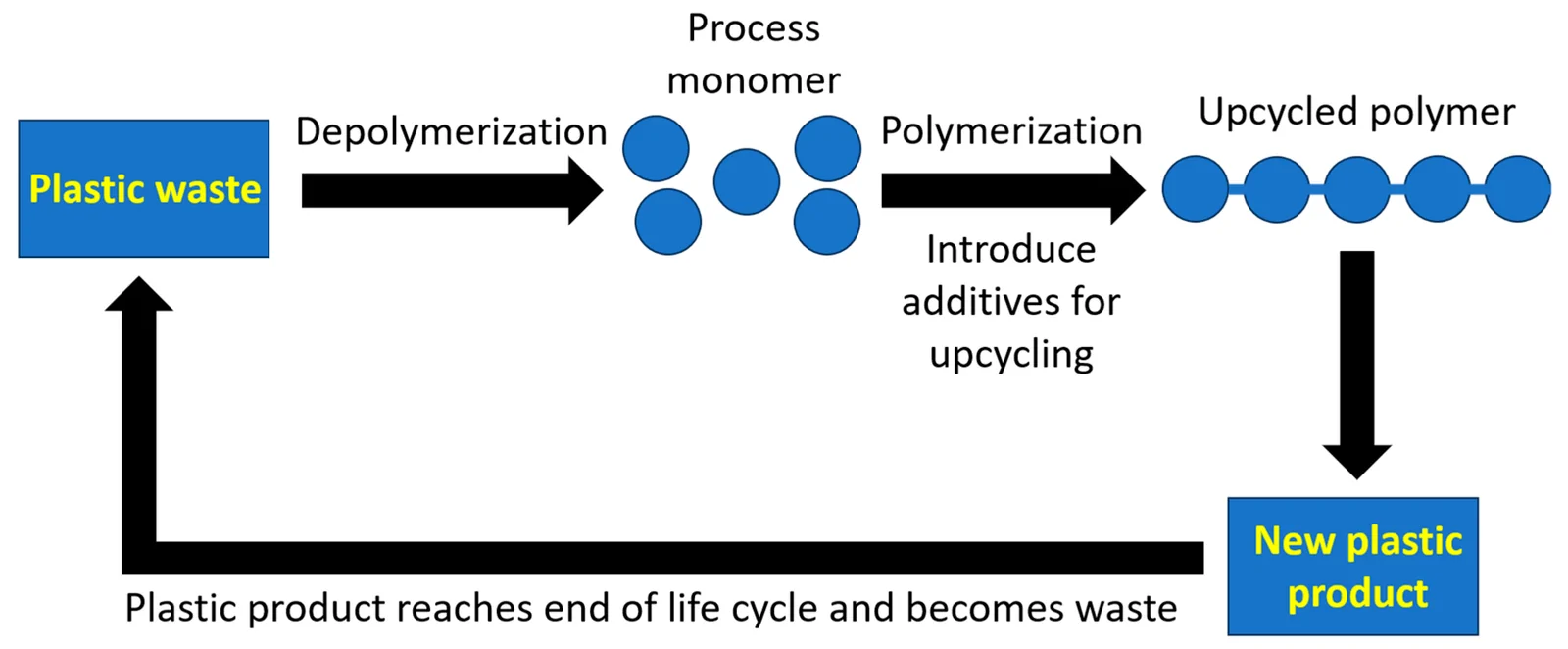
Simplified depolymerization and upcycling process diagram.

Depolymerization can be considered a fully chemical recycling process, as it focuses on the conversion of a polymer back to its monomers [191]. Depolymerization methods via solvolysis, such as methanolysis and glycolysis [192,193,194], often have a greater amount of carbon emissions and energy consumption due to the intensity of the process compared to solvent-based recycling methods like dissolution–precipitation or delamination [195]. This tradeoff is an important consideration when looking into the optimal recycling path for recycling flexible plastic waste. Based on its polymerization chemistry, PET is most suitable for depolymerization [196]. PET is particularly interesting, as it can be depolymerized with enzymes, where the enzymes target only PET and not any other polymer present. Initially, the enzymatic breakdown of PET to a monomer was not considered possible due to the crystalline structure of PET and the overall resistance to degradation [197]. However, with the introduction of hydrolases such as PETase, PET depolymerization could be achieved via enzymes under conditions close to ambient [197]. Following this discovery, enzymatic depolymerization of PET became a focus of research. The process is still the same as other depolymerization techniques in that a target polymer is depolymerized. The reclaimed monomer is then polymerized and then turned into a plastic product in a preferably closed-loop sense. Figure 7 shows a simplified depolymerization process for recycling flexible plastic waste.

Engineering enzymes specific for PET hydrolysis is one way to circumvent the biodegradation resistance seen in PET [198]. Such an enzyme was able to attack ester bonds in PET and depolymerize it to its monomer, with additional benefits from having a large surface area of PET to work with [198]. In future work, the enzyme and the reaction solvents being used could be investigated to improve efficiency, like a change from glycerol, which impedes the enzyme activity [198]. Use of enzymes for biodegradation offers a potential new pathway of depolymerization for plastic waste in the future.

Improving the depolymerization of plastic films allows for more options in recycling. PET films are heavily researched for depolymerization because of PET hydrolases. Using directed evolution on a PET hydrolase provides improvements to the depolymerization process [199]. This new type of hydrolase, DepoPETase, achieved complete depolymerization of PET waste during testing under mild temperatures [199]. This is another case of a high-performing enzyme that could be scaled up. Showing that complete depolymerization was possible, this enzyme is a viable choice for scale-up to industrial waste treatment of PET waste like PET films [199]. Having additional options for a PET hydrolase allows for more control and better recycling rates as the technology is advanced further. Use of this recycling technology on PET-containing post-consumer multilayer films may offer an additional solution to the growing amount of multilayer plastic film waste and recovery of the bound materials.

Expanding on the concept of enzyme-based depolymerization would involve optimizing it. The use of machine learning (ML) algorithms to find the best possible enzyme for depolymerizing PET films by searching for the best hydrolase worked well [200]. The new enzyme structure had a greater temperature range to work with, 30 °C to 60 °C, and higher activity in comparison to other enzymes [200]. By testing many theoretical structures at once, the best one could be found, and consideration of the system conditions was also done. The depolymerization worked well enough that it could, in theory, be used for closed-loop recycling of PET, as the collected monomer was of high enough quality [200]. A self-contained recycling loop that produces materials on par with virgin plastics is a desirable outcome for recycling plastic films.

### 5.5. Recent Developments in Plastic Film Recycling Technology

Recent research that supports plastic film recycling is highlighted in Table 7. The table includes the objective of each work, the key methods and materials used, the type of plastic waste feedstock relevant to plastic film recycling, and the key results of each work. The table is organized into a collection of innovative processes: plastic waste identification, compatibilization, solvent-targeted dissolution, delamination, and enzymatic depolymerization of PET. This list, while not exhaustive due to the volume of research that is published regarding plastic recycling and consideration for the contribution of the work to plastic film recycling, highlights recent examples that push forward film-recycling processes. For example, work done on the dissolution of a specific polymer could be applied to plastic film recycling containing said polymer, despite the published work focusing on rigid plastics instead of flexibles.

## 6. Conclusions

Advancements in the recycling of plastic film contribute to addressing the problem of plastic film waste management. Compatibilization and equipment redesigns to incorporate films have helped the mechanical recycling process use more film feedstock. Improvements to sorting systems via sensor technology and AI assistance help collect and bale film waste for feedstock use in a proper recycling process. Chemical recycling as a whole has seen great innovation, especially in the depolymerization of polymers, to enable the recycling of film waste. Solvent-based recycling processes like dissolution–precipitation and delamination have both seen the development and optimization of processes in conjunction with new solvent classes being applied to these processes. This analysis of works from recent years demonstrates the great progress achieved in plastic film recycling and its continued growth.

Plastic waste buildup is a global problem that must be dealt with. Utilization of plastic films into recycled plastics would be a good step towards reducing plastic waste buildup and the formation of a circular economy. With at least 22% of global plastic production going to flexible films as of 2023 [6,10], there is a consistent waste generation problem globally. Relevant research is constantly being published and is a step in the right direction to recycle flexible plastic waste. Other actions can also be taken to mitigate further damage. Banning single-use plastic has been a legislative movement seen in recent years to combat excessive plastic waste [63]. Globally, the United Nations has published reports on plastic pollution [15] and is holding summits to address the global plastic waste problem [227]. European, Chinese, and American legislation has also made decisions to promote a circular economy in recent years [170]. The European Union has brought several articles of legislation to reduce plastic waste and place accountability on plastic producers for plastic waste [228,229,230]. Additionally, with China banning plastic waste imports in 2018, nations like the US must be able to recycle their own plastic waste [231]. Legislation in the US, as the country with the greatest consumption of plastics [232], has been focused on introducing recycled resin content into plastic products [233] and also placing accountability on manufacturers [233,234], as in the European Union. Increased recycling efforts are, therefore, needed to meet legislative requirements in the upcoming years, and an organized response is crucial to this [235]. Meeting these requirements will help to stop further environmental damage from the overuse of plastic and unchecked plastic waste buildup.

Innovative designs are being advanced currently to provide solutions for the problem of plastic film waste. Groups like Carrot host competitions to bring out ideas and projects, such as improved sorting methods, thermal treatment of plastic films, solvent treatment of plastic films, and more [150]. Further work with plastics can be done outside of recycling as well. Increased awareness of the problems associated with waste buildup and how these problems can be addressed going forward [236]. This can also be done by changing the design of current plastics. Designing a plastic to be recyclable is important to promoting a circular economy [237]. Considerations for plastic sustainability [238], the impact of contaminants such as non-intentionally added substances (NIAS) [126], the biodegradability of plastics [239], and the effectiveness of new plastic designs meeting requirements [240] are all challenges currently being addressed. Reduction or elimination of hazardous additives that are known to cause health and environmental problems is something that can be done in the design stage of plastics [241,242]. This can be seen with work like improvements to solvents promoting energy efficiency in recovery processes [243] and recent advancements in green solvent viability [244]. Improving solvents to reduce energy consumption or be less hazardous are beneficial innovations.

Improvements and novel technologies contribute to recycling processes beginning to incorporate films as feedstock. There is still more to be done, however, before plastic films are able to truly become part of the circular economy. Sorting and identification systems have begun to have better recognition of plastic films within an MRF using artificial intelligence [101,103], odor characterization [202], and infrared spectroscopy [99,201], yet there are still complications when identifying black plastics or the structures and compositions of multilayer films. Compatibilization of mixed polymer waste shows promise for specific cases [155,156,157], but this is not suitable for a general approach to the recycling of plastic film waste. Dissolution–precipitation recycling methods [31,59,181,209,213], including STRAP [46,120,164,179,180,210], excel in the recovery of materials from both mono- and multilayer films. However, this comes at the expense of solvent and energy consumption alongside the requirements of additional solvent separation steps in a recycling process. Delamination is able to separate and collect bound materials in a multilayer film structure [10,93,144,162,163,165,183,185,187,190,219,221,222,223] with less energy and material consumption than dissolution–precipitation and no degradation to recovered solid films. Delamination work has focused heavily on metallized films in favor of inducing delamination at the weak electrostatic bonds at metal interfaces [10,93,162,163,183,190,219,221,222,223], limiting the potential of the recycling process and ignoring the potential feedstock sources of polymer-only films that are more challenging to delaminate. Research into biological depolymerization of PET [77,102,198,224,225,226] brings an interesting opportunity to recycling PET films, but is limited to PET, which makes up only an estimated 6.2% of all plastics produced globally [8].

While these new technologies promote the recycling of plastic film waste, there have still not been any breakthrough technologies to disrupt the field and show efficiency and consistency at a scale needed for mitigating the plastic film waste buildup. The application of novel solvents like switchable hydrophilicity solvents [10,163,221] or deep eutectic solvents [223,244] has interesting results in film recycling applications but are merely extensions of previous works. Furthermore, recent collections of the literature, both research articles and reviews, have oversights that need to be acknowledged. For reviews, it is very common for some recycling methods to be mislabeled or focus on a particular subset of information, which overlooks promising new developments. The best example of this is seen with plastics recycling reviews misusing terminology such as “disintegrate” for what should be dissolution when inducing delamination [29]. With articles that report on novel discoveries, there needs to be some consistency with the terms used in the plastics recycling field. Terms like “polymer separation strategies” [212] as another way to say “delamination” or the generic term of “treatment” in a title [156] for a journal article about compatibilization reduces the visibility of useful information to those that may need it. Both mislabeling and inconsistent terms should be addressed to disseminate information efficiently and effectively throughout the plastics recycling community.

Plastic film waste presents an invaluable resource that will not be going away anytime soon, so its inclusion in the circular economy is important. Research into new technologies like improved identification of waste streams, compatibilization of plastic film waste, bio-depolymerization of PET films, delamination, and solvent-targeted dissolution all offer advantages. This creates motivation for continued research and work into plastic film recycling. Additional incentives from governments to research recycling technologies can promote recycling and mitigate further negative impacts caused by plastic film waste buildup. Through advancements in recycling technologies, it is only a matter of time before such a large source of plastic is reclaimed and not ignored.

## Figures and Tables

**Table 1:** Global Plastic Generation Statistics.

Year	2019 [4]	2021 [4]	2023	2024
Production Capacity	252 MMT	268 MMT	414 MMT [8]	430 MMT [5]
Production Volume	215 MMT	230 MMT	414 MMT [8]	430 MMT
Recycling Capacity *	23 MMT	25 MMT	37 MMT [8]	41 MMT
Single-Use Plastic Consumption	133 MMT	139 MMT	>150 MMT [9]	172 MMT [5,7]
Flexible Plastic	55% Single-Use Plastic	57% Single-Use Plastic	>55% Single-Use Plastic [4]	>55% Single-Use Plastic [4]

* Recycling capacity for polyethylene, polypropylene, polystyrene, and polyethylene terephthalate.

**Table 2:** All Plastic Waste From 1950 to 2015 [18].

**All Plastic Waste from 1950 to 2015**	
Total Plastic Production	8300 MMT
Plastics In Use	2500 MMT
Waste Plastic	5800 MMT
**Waste Plastic Metrics**	
Landfilled and Polluted Waste	4900 MMT
Incinerated Waste	800 MMT
Recycled Waste	600 MMT

**Table 3:** Common Polymers and Materials Used in Multilayer Films [35,36].

Function	Materials
Heat Sealing	PE, PP, PET, PA, PU
Moisture/Gas/Aroma Barrier	PE, PP, PET, PS, PVC, PA, EVOH, PVDC, Aluminum Foil
Mechanical Strength	PET, PVC, PA, Paper/Paperboard
Printability	PS
Adhesive	EVA, PU
Abrasion Resistance	PVDC, Paper/Paperboard
Light Barrier	Aluminum Foil

Acronyms used: PE = polyethylene; PP = polypropylene; PET = polyethylene terephthalate; PA = polyamide; PU = polyurethane; PS = polystyrene; PVC = polyvinyl chloride; EVOH = ethyl vinyl alcohol; PVDC = polyvinylidene chloride; EVA = ethylene–vinyl acetate.

**Table 4:** Comparison of Polyethylene to Other Materials Used in Packaging.

Material	Young’s Modulus (GPa)	Elongation (%Strain)	Density (kg/m^3^)	Cost (USD/kg)
Polyethylene	1.07–1.09 [37]	11.2–12.9 [37]	925–965 [37]	0.10–1.65 [39]
Aluminum Foil	68–82 [38]	0.01–0.44 [38]	2500–2900 [38]	2.76–4.41 [40]
Glass	70 [42]	N/A [42]	2500 [42]	0.58 [41]

**Table 5:** Common Additives in Plastic Films [54].

Additive Class	Additive Weight %	Common Additives
Plasticizers	10–70%	Paraffins, phthalic esters, acetyltributyl citrate
Slip agents	0.1–3%	Erucamide, fatty acid esters, and waxes
Antioxidants	0.05–3%	Bisphenol A, nonylphenol compounds
Flame Retardants	3% to 25%	Paraffins, boric acid, polybrominated diphenyl ethers
Fillers	0% to 50%	Glass, talc, calcium carbonate

**Table 6:** Plastic Recycling Methods.

Treatment Method	Process	Plastics Treated
Mechanical Recycling	Grinding [20,68,69] Granulation [68,69,70] Extrusion [68,69,71,72,73]	HIPS, LDPE, HDPE, and PET [20] Single polymer waste streams [70] PET, HDPE, LDPE, PP, and PVC [69]
Chemical Recycling	Dissolution [68,74] Depolymerization [75,76,77,78,79,80,81,82,83] Pyrolysis [75,84,85,86,87,88] and Hydrocracking [89,90,91] Gasification [80,86,92,93,94,95,96,97]	PS, PP, HDPE, and ABS [68,74] PET [77,80,82], PU [80],PA [80], PC [80], PA6 [83] and PLA [80] MPW [75], and contaminated plastics [76] PE [75,84], PP [84], PS [84], PET [84], PVC [84], contaminated plastics [85] PE, PP, PS, and PET [89] PE [92], PET [92], PP [92], PS [92], MPW [92], and MPP [93]
Energy Recovery	Valorization [62]	Plastic Waste [62]

Acronyms used in table: HIPS = high impact polystyrene; ABS = acrylonitrile–butadiene–styrene; PU = polyurethane; PC = polycarbonate; PLA = poly(lactic) acid; MPW = mixed plastic waste; MPP = metallized plastic packaging.

**Table 7:** Summary of Recent Advancements in Emerging Recycling Methods.

Objective	Methodologies and Materials Used in Work	Recycling Feedstock	Products and Key Results
**Innovative Recycling Processes**			
Wash post-consumer film to restore to food grade [150]	Supercritical CO_2_ [150]	Polyolefin films [150]	Washed films are considered food-grade [150]
Sort monolayer and multilayer films [150]	BOSS-2D process [150]	Household flexible film [150]	95% Separation of films [150]
Solvent-based washing system for films [150]	CleanBlueTech process [150]	PE [150]	Waterless, 30% energy of regular sorting [150]
Solvent-targeted polymer recovery [150]	Polymer-specific solvents [150]	Plastic films [150]	Resin recovery and recoverable solvents [150]
**Plastic Identification**			
Characterization of post-consumer waste [99]	Near-infrared spectroscopy [99]	Multilayer packaging [99]	Achieved 96% identification accuracy [99]
Characterization of post-consumer plastic films [201]	TGA, FTIR, DSC, and ultimate and proximate analysis	Post-consumer plastic film waste [201]	Compared sorting efficiency between manual and automatic [201]
Odor-based characterization for plastic films [202]	Gas chromatography [202]	Polyolefin films [202]	Characterized 58 odor-producing contaminants [202]
Use of near-infrared spectroscopy for waste characterization [203]	Near-infrared spectroscopy [203]	PP, LDPE, PET [203]	Near-infrared spectroscopy can identify multilayer compositions [203]
Assessment of Performance and Challenges in Use of Commercial Automated Sorting Technology for Plastic Waste [101]	Automated sorting via artificial intelligence [101]	Municipal Waste Streams [101]	Film sorting is still poorly done and requires additional work [101]
Recent Developments in Technology for Sorting Plastic for Recycling: The Emergence of Artificial Intelligence and the Rise of the Robots [103]	Artificial intelligence for automatic identification [103]	Municipal Waste Streams [103]	Improvements to film sorting, challenges with dark plastics and multilayer films [103]
Accurate characterization of mixed plastic waste using ATR-FTIR spectroscopy and machine learning methods [204]	ATR-FTIR and machine learning [204]	Municipal Waste Streams [204]	≥92.5% correct identification accuracy via algorithmic methods [204]
AI-enabled spectral classification of plastic resins from E-waste via laser-induced breakdown spectroscopy for advanced sorting applications [205]	Spectral characterization with artificial intelligence [205]	E-waste [205]	≥96% correct characterization of plastic resins in E-waste [205]
**Compatibilization**			
Treatment of starch–polymer mix [156]	Grafted polycaprolactone, maleic anhydride and glycidyl methacrylate [156]	Thermoplastic starch and PLA [156]	Optimized mixture ratios to achieve targeted properties [156]
Compatibilization of PE film for reactive extrusion [155]	PE grafted with maleic anhydride [155]	PE film [155]	Compatibilized LDPE film for reuse [155]
Investigation into PE/EVOH compatibilization [157]	Two-photon microscopy and ODIN-isocyanate [157]	PE and EVOH [157]	Compatibilization was achieved and ODIN proved viable as a compatibilizer [157]
Recycling Polyethylene/Polyamide Multilayer Films with Poly(isoprene-g-Maleic Anhydride) Compatibilizer [206]	PI-g-MA compatibilizer [206]	PE and PA [206]	Successful compatibilization [206]
Household Post-Consumer Flexible Plastic Recycling—The Significance of Compatibilizers and Reinforcing Fillers	Maleic anhydride [207]	Post-consumer flexible plastic [207]	Successful compatibilization with respect to mechanical properties [207]
Topological universal dynamic compatibilization enhances recycling of mixed plastics [132]	Topological crosslinking agent [132]	Mixed polar and apolar plastics [132]	Successful upcycling of mixed plastic waste via reactive extrusion [132]
Mechanical recycling of multiphase contaminated plastic waste via physical compatibilization: a study on rheological, morphological and mechanical properties [208]	Propylene–ethylene elastomer and ethylene–octene copolymer [208]	PE/PP blends [208]	Ethylene–octene copolymer proves a viable compatibilizer for PE/PP blends [208]
**Dissolution–Precipitation**			
Recovery of materials from multilayer packaging foil [162]	Ethyl acetate, ethanol, toluene, acetone, acetic acid, and dimethyl formamide [162]	PE and aluminum blend [162]	Successful recovery of aluminum and PE [162]
Recycling of post-consumer plastic multilayer waste [181]	Xylene, Isopropanol, and hydro pulping [181]	Blend of LDPE, Paperboard, and aluminum [181]	Successfully recovered all components [181]
Creation of new solvent affinity calculation [169]	Solvent and polymer databases [169]	PE and EVOH [169]	COSMO-RS as new solubility calculation [169]
Polyamide 6 targeted dissolution [209]	Dimethyl sulfoxide [209]	PA 6 [209]	Recovered PA 6 from mixed plastic waste [209]
Reduction of antisolvent use for multilayer plastic recycling [210]	Toluene cyclohexanone, 1,4-dioxane, triethylamine, and acetone [210]	PE, PET, Glycol-modified PET, EVA, EVOH [210]	Antisolvent consumption reduced through temperature-controlled dissolution to reduce cost [210]
Use of new solvents to reduce energy of solvent recovery post-treatment [163]	Switchable-hydrophilicity solvents [163]	PET, PP, PE, EVOH, PS, aluminum blends [163]	New solvent type successful in recovering polymers [163]
Recycling of post-consumer aseptic multilayer packaging [211]	Xylene, hexane, ethyl acetate, and non-polar solvents [211]	PE and aluminum blend [211]	Successfully separated and partially recovered PE [211]
Life cycle assessment of STRAP variations for recycling multilayer films [59]	Toluene, acetone, 1-propanol, dimethyl sulfoxide, tetrahydrofuran [59]	PE, EVOH, PET, EVA and PETG [59]	Temperature-driven precipitation reduces carbon emissions compared to antisolvents [59]
Large-scale computational polymer solubility predictions and applications to dissolution-based plastic recycling [212]	Molecular dynamics simulations [212]	Polymers modeled in software [212]	Provides new solvent-solute combinations for recycling applications [212]
Recycling of a post-industrial printed multilayer plastic film containing polyurethane inks by solvent-targeted recovery and precipitation [120]	Gamma-valerolactone [120]	Multilayer film [120]	Removal of colorants and Technoeconomic analysis [120]
Dissolution recycling for recovery of polypropylene and glass fibers [160]	xylene [160]	PP and glass blend [160]	Recovery of both materials via precipitation and filtration [160]
Periodic alternation between precipitation and dissolution of camphor solid film in an ethanol solution [158]	Ethanol [158]	Camphor and plastic film [158]	New mathematical model to predict thermal diffusion phenomenon [158]
Cast Film Production with Polyethylene Recycled from a Post-Industrial Printed Multilayer Film by Solvent-Targeted Recovery and Precipitation [159]	STRAP procedure optimization [159]	Multilayer plastic films [159]	High-quality reprocessed materials following STRAP process [159]
Development of a solvent-based recycling process for agricultural film [119]	Xylene and limonene [119]	LDPE agriculture film waste [119]	98% purity of recovered LDPE [119]
Characterization of the unknown chemical composition of a commercial biodegradable agricultural plastic mulch film using complementary spectrometric and spectroscopic techniques [127]	Compatible solvents for dissolution of PLA [127]	PLA/PBAT agricultural mulch film [127]	Report on 83 additional substances not detected by common characterization methods on initial film waste [127]
A solvent-targeted recovery and precipitation scheme for the recycling of up to ten polymers from post-industrial mixed plastic waste [180]	Solvent selection with a computational model [180]	10-polymer mixed waste system [180]	89% or higher recovery rate of all polymers [180]
Pigment removal from reverse-printed laminated flexible films by solvent-targeted recovery and precipitation [164]	Activated carbon adsorbent in addition to solvents [164]	Multilayer films [164]	Activated carbon proved highly successful in pigment removal [164]
Solvent-based dissolution–precipitation of waste polyethylene terephthalate: economic and environmental performance metrics [213]	Technoeconomic analysis and life cycle assessment of dissolution–precipitation [213]	Post-consumer PET [213]	Dissolution–precipitation is better environmentally than virgin resin products and is economically feasible [213]
Experimental Tests on Solvent Solutions for the Recycling of Coated Polypropylene in Food Packaging [214]	KOH [214]	PP/aluminum film [214]	Recovery of both PP and solvent to near 100% [214]
Reclaiming Polyethylene from Multilayer Plastic Packaging by Separating Oxygen Barrier Material Using Solvent-Based Selective Dissolution: Recycling Polyethylene for Property Characterization [215]	Formic acid [215]	5-layer multilayer film [215]	Recovery of EVOH and polyolefins successful following hydrolysis of maleic anhydride-grafted LLDPE [215]
Aqueous Dissolution and Recovery of Poly(Vinyl Alcohol) in Multilayer Film [216]	Water [216]	Poly(Vinyl Alcohol) multilayer film [216]	PVOH was dissolved and recovered at near-virgin resin quality chemically [216]
Production of high-quality polyethylene (PE) films from post-consumer shrink wrap with solvent targeted recovery and precipitation (STRAP) [182]	Xylenes [182]	Shrink wrap [182]	Shrink wrap is a viable feedstock for the STRAP process [182]
**Delamination**			
Targeted glycolysis of adhesive layer in multilayer packaging [165]	Diols [165]	PA/PU/PP blend [165]	Delamination was successful and only targeted PU [165]
Kinetic analysis of delamination by carboxylic acids [187]	Aspen Plus^®^ [187]	PET/PA and PET/PE [187]	Optimized delamination conditions of select polymer blends [187]
Promotion of delamination by microperforation [190]	Caustic soda [190]	PET/aluminum blend [190]	Microperforation improved the delamination process [190]
Packaging design using reversible cross-linked adhesive to promote delamination [217]	PU adhesive and dimethylsulfoxide [217]	PE/PET, PET/aluminum, and PE/aluminum blends [217]	Promoted delamination through adhesive via Diels-Alder equilibrium [217]
Recycling of multilayer packaging by separation of heterogenous layers [218]	Acetone and supercritical ethanol [218]	PE/PET/aluminum blend [218]	Successful separation of layers allowing for recycling of each material [218]
Delamination through dissolution of PU adhesive [219]	Carboxylic acids [219]	PE, PET, and PP [219]	PU was successfully dissolved and delamination was achieved [219]
Delamination of flexible plastic waste by high heat treatment [93]	High temperatures 275 °C to 700 °C [93]	PET/PP/aluminum blend [93]	High-purity aluminum was recovered at the cost of the polymers [93]
Patent for separation of multilayer film packaging [220]	Polyvinyl alcohol adhesive, acids, organic solvents [220]	PET aluminum and paperboard blend [220]	Patented delamination process [220]
Recycling of multilayer packaging waste with switchable anionic surfactants [221]	Switchable Anionic Solvents (SAS) [221]	Metallized films [221]	Recovery of both polymers and metal materials [221]
Chemical Delamination Applicable to a Low-Energy Recycling Process of Photovoltaic Modules [222]	Toluene, THF, U 60002, Cyclohexane [222]	Solar Panels [222]	Poor material recovery rate due to strong solvent usage [222]
Delamination and Evaluation of Multilayer PE/Al/PET Packaging Waste Separated Using a Hydrophobic Deep Eutectic Solvent [223]	Hydrophobic Deep Eutectic Solvent [223]	PE/AL/PET film [223]	Good separation and material recovery of all layers present [223]
Recycling of Multilayer Flexible Packaging Waste Through Delamination with Recoverable Switchable Hydrophilicity Solvents [10]	Switchable Hydrophilicity Solvents [10]	Metallized films [10]	Achieved delamination and recovery of materials present in multilayer films [10]
Mechanical Properties of Polymers Recovered from Multilayer Food Packaging by Nitric Acid [184]	Nitric Acid [184]	Multilayer film [184]	Mechanical values lowered after recovery [184]
Physical–chemical hybrid process for separating all raw materials from the waste of liquid crystal display screens of smartphones [185]	Ethyl acetate and ultrapure water [185]	Liquid Crystal Displays [185]	Polymers delaminated at one hour [185]
Flash separation and recovery of each component from waste photovoltaic modules [144]	Thermal expansion of layers [144]	Photovoltaic modules [144]	98% recovery rate of separated components [144]
Coupling of chemical deconstruction and pyrolysis to upcycle metallized multilayer plastic films [145]	Targeted depolymerization of PET with ammonia to recover separated layers [145]	Metallized multilayer film [145]	Reduced contamination and high recovery of materials [145]
Delamination of Multilayer Plastic Films to Recover Polyethylene with Favorable Mechanical Properties [33]	Formic acid to dissolve polyurethane adhesive tie layers [33]	Multilayer film with majority PE [33]	Successful separation and recovery of PE and PET as solid film [33]
**Enzymatic Depolymerization of PET Films**			
Enzymatic hydrolysis of PET [198]	PET hydrolase Cut190 enzyme [198]	PET and PE furanoate [198]	Enzyme was capable of biodegradation of PET by hydrolysis [198]
Optimization of depolymerization by machine learning [200]	PET hydrolase FAST-PETase [200]	PET films [200]	Achieved closed-loop recycling process for PET [200]
Direct evolution to produce a better hydrolase for depolymerization [199]	DepoPETase [199]	PET films [199]	Hydrolase capable of reaching industrial-scale application [199]
Enzymatic depolymerization of polyester: Foaming as a pretreatment to increase specific surface area [102]	Leaf compost-cutinase (LCC-ICCG) enzymes [102]	PET films [102]	Improvements to depolymerization rates [224]
Complete Enzymatic Depolymerization of Polyethylene Terephthalate (PET) Plastic Using a Saccharomyces Cerevisiae-Based Whole-Cell Biocatalyst [224]	Saccharomyces cerevisiae-based whole-cell biocatalyst [224]	PET Films [224]	Improved depolymerization at 30 °C [224]
Efficient Bioprocess for Mixed PET Waste Depolymerization Using Crude Cutinase [225]	Fast PETase, LCC and LCCICCG [225]	Mixed PET film waste [225]	Successful depolymerization in mixed waste system [225]
Efficient Recycling of PET-PE Multilayer Packaging Materials Based on Enzymatic Depolymerization of PET [77]	Polyester hydrolases [77]	PET multilayer film [77]	Depolymerization in mixed post-consumer waste [77]
One-Pot Depolymerization of Mixed Plastics Using a Dual Enzyme System [226]	Polyester hydrolase PES-H1 FY and (poly)urethanase UMG-SP-2 [226]	Mixed PET waste [226]	Successful depolymerization without sorting pre-depolymerization [226]

## Data Availability

This is a review of published literature. No original data were generated.
